# Material-Dependent Formation and Degradation of Bone Matrix—Comparison of Two Cryogels

**DOI:** 10.3390/bioengineering7020052

**Published:** 2020-06-05

**Authors:** Weidong Weng, Victor Häussling, Romina H. Aspera-Werz, Fabian Springer, Helen Rinderknecht, Bianca Braun, Markus A. Küper, Andreas K. Nussler, Sabrina Ehnert

**Affiliations:** 1 Siegfried Weller Research Institute, BG Unfallklinik Tübingen, Department of Trauma and Reconstructive Surgery, University of Tübingen, Schnarrenbergstr. 95, D-72076 Tübingen, Germany; wengweidong5657@gmail.com (W.W.); victor.haeussling@student.uni-tuebingen.de (V.H.); rominaaspera@hotmail.com (R.H.A.-W.); helen.rinderknecht@student.uni-tuebingen.de (H.R.); bibi.braun@gmx.de (B.B.); mkueper@bgu-tuebingen.de (M.A.K.); sabrina.ehnert@gmail.com (S.E.); 2 Department of Diagnostic and Interventional Radiology, University of Tübingen, Hoppe-Seyler-Str. 3, D-72076 Tübingen, Germany; fabian.springer@med.uni-tuebingen.de; 3 Department of Radiology, BG Unfallklinik Tübingen, Schnarrenbergstr. 95, D-72076 Tübingen, Germany

**Keywords:** bone tissue engineering, cryogel, bone cells, 3D-culture, scaffold, platelet-rich plasma (PRP), gelatin, matrix metabolism

## Abstract

Cryogels represent ideal carriers for bone tissue engineering. We recently described the osteogenic potential of cryogels with different protein additives, e.g., platelet-rich plasma (PRP). However, these scaffolds raised concerns as different toxic substances are required for their preparation. Therefore, we developed another gelatin (GEL)-based cryogel. This study aimed to compare the two scaffolds regarding their physical characteristics and their influence on osteogenic and osteoclastic cells. Compared to the PRP scaffolds, GEL scaffolds had both larger pores and thicker walls, resulting in a lower connective density. PRP scaffolds, with crystalized calcium phosphates on the surface, were significantly stiffer but less mineralized than GEL scaffolds with hydroxyapatite incorporated within the matrix. The GEL scaffolds favored adherence and proliferation of the osteogenic SCP-1 and SaOS-2 cells. Macrophage colony-stimulating factor (M-CSF) and osteoprotegerin (OPG) levels seemed to be induced by GEL scaffolds. Levels of other osteoblast and osteoclast markers were comparable between the two scaffolds. After 14 days, mineral content and stiffness of the cryogels were increased by SCP-1 and SaOS-2 cells, especially of PRP scaffolds. THP-1 cell-derived osteoclastic cells only reduced mineral content and stiffness of PRP cryogels. In summary, both scaffolds present powerful advantages; however, the possibility to altered mineral content and stiffness may be decisive when it comes to using PRP or GEL scaffolds for bone tissue engineering.

## 1. Introduction

The treatment of large bone defects remains a challenge for orthopedics and trauma surgeons [1], as it requires both fixation of bone fragments in an anatomically correct position and osseous bridging of the gap [2]. Surgical techniques, e.g., the Masquelet technique, or the Ilizarov fixator, established to repair large bone defects, require vast amounts of biologically active bone material [3,4]. The availability of bone tissue is, therefore, a critical issue [5]. Patients’ own bone for autologous transplantation is limited and allogeneic transplantation of bone tissue from donors is always accompanied by immunological risks. Alloplastic synthetic bone filling materials may be used to fill the defect area. However, to compensate for the lack of biologic activity, these synthetic bone filling materials are often mixed with platelet-rich plasma (PRP), bone marrow concentrate, or reaming irrigation aspirate of the recipient to provide osteogenic precursor cells to the fracture [6]. Tissue engineering aims to solve this issue by producing biologically active bone material for transplantation, which ideally consists of the patients’ own cells on a scaffold that mimics the extracellular bone matrix [7].

In bone tissue engineering, 3D carriers mimicking the three-dimensional environment of bone favor the maturation and function of bone cells applied on these carriers [8]. Therefore, the premise evolved that 3D cultures are more beneficial for cellular differentiation than 2D cultures [9]. But 3D cultures also raise new challenges, i.e., proper adjustment of the physical characteristics of the 3D matrix to the human physiology, uniform cell seeding on, or adequate nutrient supply within the scaffold [10]. Regarding scaffold architecture, pore size and shape, wall thickness, porosity, and stiffness are essential [11]. Pore structure and porosity not only affect cell attachment on the scaffold and cell infiltration into the scaffold but also the nutritional supply and metabolite removal, a factor defined by medium diffusion and characterized by the permeability of the scaffold. The scaffold’s stiffness may affect differentiation of the applied progenitor cells—while scaffolds with low stiffness favor adipogenic and chondrogenic differentiation, scaffolds with higher stiffness favor osteogenic differentiation [12]. At the same time scaffolds for bone tissue engineering ideally sustain some flexibility to pass on mechanical stimuli to the cells [13], as this is known to favor osteogenic differentiation [14].

These examples show that the choice of scaffold strongly affects the success of the 3D culture. Currently, numerous raw materials and production processes are being tested, all providing specific advantages and disadvantages [15,16]. Among the huge number of scaffolds available, cryogels stand out because of their porous structure, which effectively mimics the structure of cancellous bone [17,18]. Various monomers and cross-linkers can serve as building blocks for cryogels—for an overview, see Table 1.

Besides the huge variability that these different monomers and cross-linkers provide, cryogels are easy and cheap to produce and can be generated in different shapes. The choice of polymer and cross-linker, in combination with the freezing temperature, affect pore size and stiffness of the gels [17]. 2-Hydroxyethyl methacrylate (HEMA) is a commonly used monomer for the generation of cryogels. It is often polymerized with methyl-methacrylate or N,N-methylene(bis)acrylamide (BAAm) as cross-linkers. The resulting cryogels are frequently used as scaffolds for in vitro studies or as carriers for tissue engineering approaches [19], especially as protein structures (e.g., collagen, gelatin, or PRP) can be covalently linked to the polymer using glutaraldehyde [11]. HEMA-based cryogels have been shown to improve the healing of large calvarial bone defects in rabbits [20]. Although, the known toxicity of the individual building blocks, e.g., HEMA, BAAm, ammonium persulfate (APS), or N,N,N,N-tetramethyl-ethylenediamine (TEMED), is no longer given when present in the interconnected solid form as polymerized material [21], discussions on the suitability of such cryogels for tissue engineering purposes and on the applicability in humans arise every now and then. Several studies have shown that with an adequate cleaning protocol, residual toxins and impurities can effectively be removed, such that cells can successfully attach to and grow on these cryogels [7,22]. Considering cryogels’ in vivo application, biological persistence is of special interest. HEMA based cryogel may exhibit genotoxic effects on the surrounding tissue, when mechanically degraded [23]. To minimize possible toxicity, simplified cryogels with only a limited amount of building blocks were generated. Cryogels mainly composed of components naturally occurring in the target tissue, as collagen or gelatin for bone, are supposed to be degraded with time in vivo, allowing complete remodeling of the replaced tissue [11]. However, changes in the material composition and the freezing process are well known to strongly affect the cryogels’ characteristics [7,11].

Therefore, in this study, we aimed to characterize structural features (stiffness, porosity, pore size, and shape) of HEMA- and gelatin-based cryogels with regard to mimicking bone architecture. Furthermore, we aimed at defining the compatibility of these cryogels with different types of bone cells (mesenchymal stem cells, osteoblastic, and osteoclastic cells) and investigate the cryogels effect on the formation and degradation of bone matrix.

## 2. Materials and Methods

Chemicals and reagents were obtained from Carl Roth (Karlsruhe, Germany) or Sigma–Aldrich/Merck (Darmstadt, Germany). The culture medium and its supplements were obtained from Sigma–Aldrich/Merck.

### 2.1. Scaffold Manufacturing and Sterilization

Scaffolds were manufactured by the so-called cryogel-technique. In this technique, monomers are polymerized under frozen conditions, such that the formed ice crystals serve as space holders for the pores to be formed. Freezing temperature and speed affect the resulting pore size and shape [11].

PRP scaffolds were generated as previously described [7]. Briefly, PRP was prepared by centrifugation (1000× *g*, 10 min) of EDTA blood from healthy volunteers (pool of ≥ 5 donors). An aqueous solution containing 16.0% pHEMA, 0.3% BAAm, and 0.25 g/L PRP was carefully mixed and cooled on ice. After 30 min, di-sodium hydrogen phosphate buffer was added to obtain a final solution of 0.3 M. Immediately after adding 0.1% glutaraldehyde, 0.2% APS, and 0.2% TEMED, the reaction solution was mixed, distributed into polystyrene casting molds (2 mL per mold), and frozen at −18 °C for, at least, overnight. The formed matrix was deep-frozen for 1 h at −80 °C to ease slicing with a razor blade. The obtained scaffolds had a uniform height of 3 mm and a diameter of 6 mm. The HEMA based scaffolds were immediately transferred to a 1 M CaCl_2_ solution to facilitate crystallization of calcium phosphate (hydroxyapatite). After 24 h, the CaCl_2_ solution was carefully aspirated, and the scaffolds were washed with phosphate buffered saline (PBS) for 15 min.

For generating gelatin (GEL) scaffolds, pre-cooled (30 min on ice) gelatin (cold water fish) solution and hydroxyapatite solution were mixed to obtain final concentrations of 4.8% and 10%, respectively. Immediately after adding 1% glutaraldehyde, the homogenous reaction mixture was distributed into polystyrene casting molds (2 mL per casting mold) and frozen at −18 °C for, at least, overnight. After 1 h at −80 °C, the deep-frozen matrix was sliced with a razor blade. The resulting scaffolds had a uniform height of 3 mm and a diameter of 6 mm and were washed once with PBS for 15 min.

For sterilization and to remove unreacted compounds (possible toxins), PBS was thoroughly aspirated from the scaffolds. Scaffolds were covered with 70% ethanol and incubated for, at least, 12 h with agitation. After three washing steps with PBS (1, 6, and 12 h), the sterilized scaffolds were incubated in a culture medium (48 h, 37 °C, 5% CO_2_, humidified atmosphere) for pre-conditioning and as sterility control.

### 2.2. Physical Characterization of Scaffolds

#### 2.2.1. Pore Structure

**Figure 1:**
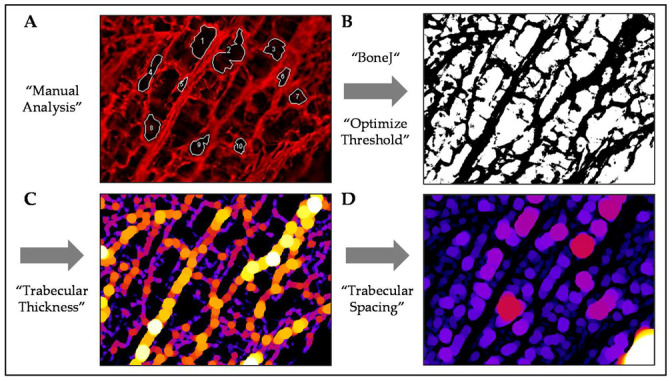
Analysis of the pore structure using the ImageJ software. (**A**) Fluorescent image of a sulforhodamine B (SRB) stained platelet-rich plasma (PRP) scaffold (100× magnification). Manual marking of the pores using the “polygon selection” tool will provide pore area, mean diameter, and circularity. (**B**) 8-bit binary image after optimizing the threshold with the “BoneJ” plugin. This allows automated analysis of factors, such as (**C**) “trabecular thickness” and (**D**) “trabecular spacing”.

Organic matrix components of the scaffolds were stained with sulforhodamine B (SRB) to visualize the scaffold structure. Briefly, scaffolds were covered with 0.08% SRB in 1% acetic acid. After 30 min (protected from light), SRB was aspirated, and scaffolds were excessively washed with 1% acetic acid [21]. SRB, bound to the scaffold membranes, emitted a red fluorescent signal, which was detected with a fluorescence microscope (Evos Fl, Thermo Fisher Scientific, Karlsruhe, GER). Fluorescent images were analyzed with the ImageJ software (NIH, Bethesda, Rockville, MD, USA). Pore size and shape were determined manually with the help of the “polygon selection” tool (Figure 1A). Per scaffold, 10 representative pores of 3 images were measured by two independent investigators in a blinded fashion. More detailed analyses of the images were performed using the “BoneJ” plugins [24] (Figure 1B–D).

#### 2.2.2. Porosity and Water Uptake Rate

The porosity and water uptake rate were determined using the scaffolds’ wet and dry weight, as described before [7]. Briefly, by using an analytical balance, the dry (scaffolds overnight in a desiccator) and wet (scaffolds immerse in water for 1 h) weight of the scaffolds were measured.

(1) $$\mathrm{porosity}\;\left[\%\right]=\frac{\left(\mathrm{scaffold}\;\mathrm{wet}\;\mathrm{weight}\;\left[g\right]-\mathrm{scaffold}\;\mathrm{dry}\;\mathrm{weight}\;\left[g\right]\right)}{\mathrm{scaffold}\;\mathrm{wet}\;\mathrm{weight}\;\left[g\right]}\times 100$$

(2) $$\mathrm{water}\;\mathrm{uptake}\;\mathrm{rate}\;\left[1\right]=\frac{\left(\mathrm{scaffold}\;\mathrm{wet}\;\mathrm{weight}\;\left[g\right]-\;\mathrm{scaffold}\;\mathrm{dry}\;\mathrm{weight}\;\left[g\right]\right)}{\mathrm{scaffold}\;\mathrm{dry}\;\mathrm{weight}\;\left[g\right]}$$

#### 2.2.3. Permeability

The permeability of the scaffold was calculated as recently published [25]. Briefly, a scaffold was placed (press-fit) into a tube. A stable hydrostatic pressure was applied to the top surface of the scaffold. Based on the water flow through the scaffold, the permeability was calculated based on Darcy’s law:

(3) $$\begin{array}{l} \mathrm{permeability}\;\left({\mathrm{cm}}^{2}\right)\\ =\frac{\mathrm{viscosity}\;\mathrm{water}\;\left(\mathrm{Pa}\cdot s\right)\times \frac{\mathrm{water}\;\mathrm{passed}\;\mathrm{through}\;\mathrm{the}\;\mathrm{scaffold}\;({\mathrm{cm}}^{3})}{\mathrm{time}\;\left(s\right)}}{\frac{\mathrm{cross}-\mathrm{sectional}\;\mathrm{area}\;\mathrm{of}\;\mathrm{the}\;\mathrm{scaffold}\;\left({\mathrm{cm}}^{2}\right)\;\times \;\Delta P\;\left(\mathrm{Pa}\right)}{\mathrm{height}\;\mathrm{of}\;\mathrm{the}\;\mathrm{scaffold}\;\left(\mathrm{cm}\right)}} \end{array}$$

At room temperature, the viscosity of water was assumed to be 9.5 × 10^−4^ Pa·s. The cross-sectional area of the scaffold was 0.785 cm^2^, and the height of the scaffold was 0.3 cm.

To determine the volume of water that passed through the scaffold per minute (60 s), the water was collected and weighed (g) using an analytical balance. Division by the specific density of water (0.997 g/cm^3^) gave the required volume in cm^3^.

The pressure change along the scaffold (ΔP) was calculated as follows:

(4) $$\Delta P\;\left(\mathrm{Pa}\right)=\frac{\mathrm{density}\;\mathrm{water}\;\left(g/{\mathrm{cm}}^{3}\right)\times \;\mathrm{scaffold}\;\mathrm{volume}\;\left({\mathrm{cm}}^{3}\right)\;}{\mathrm{gravitational}\;\mathrm{force}\;\left(m/s^{2}\right)}$$

At room temperature, the specific density of water was 0.997 g/cm^3^. The gravitational force was ~9.8 m/s^2^. The scaffold volume was determined to be 0.236 cm^3^ by multiplying the height of the scaffold (0.3 cm) with its cross-sectional area (0.785 cm^2^). The resulting ΔP was 2.396 × 10^−5^ Pa.

#### 2.2.4. Mineral Content

The mineral content of the scaffolds was determined using quantitative computer tomographic (CT) scans using a clinical 128-slice high-end CT scanner (SOMATOM Definition Edge, Siemens Healthineers, Erlangen, Germany). Specimen were scanned with 80kV tube voltage, 500 mAs effective tube current, acquisition 16 mm × 0.3 mm with 0.4 mm slice thickness, and a pitch of 0.4. Images were reconstructed using an edge enhancing very sharp reconstruction kernel (V80u) with iterative image reconstruction (SAFIRE, Siemens Healthineers, Erlangen, Germany). Obtained DICOM images were imported into the ImageJ software using the “DICOM sort” plugin. The resulting stack was cropped to show the area of interest. From each scaffold, the mean grey values were determined and normalized to the reference block (Phantom EFP-06-96). Furthermore, using the “3D viewer” plugin, a 3D reconstruction of the scaffolds was generated.

#### 2.2.5. Matrix Stiffness

Young’s modulus was used to calculate the scaffold stiffness [25]. Using a ZwickiLine Z 2.5TN (Zwick GmbH and Co. KG, Ulm, Germany) material testing machine, scaffolds were compressed four times uniaxially by 10% (speed = 5 mm/min) of the original height. An Xforce HP 5N sensor measured the required load in real-time. The resulting load-deformation curve was translated into a stress–strain curve by using the height and area of the uncompressed scaffold. Young’s modulus in the region of linear elastic deformation was calculated as follows [26]:

$${\mathrm{Young}}^{\prime }s\;\mathrm{modulus}\;\left[\mathrm{MPa}\right]=\frac{applied\;force\;\left[N\right]\times initial\;scaffold\;height\;\left[mm\right]}{\;area\;of\;the\;scaffold\;\left[{\mathrm{mm}}^{2}\right]\times change\;in\;height\;\left[mm\right]}$$

### 2.3. Cell Culture

#### 2.3.1. Cell Lines

THP-1 cells (DSMZ) were used as osteoclastic precursor cells. Cells were cultivated as a suspension cell culture in RPMI 1640 Medium supplemented with 5% FCS (37 °C, 5% CO_2_, humidified atmosphere). Cell density was kept between 2 × 10^5^ and 1 × 10^6^ cells/ml for expansion. The medium was changed twice a week.

Immortalized bone marrow-derived mesenchymal stem cell line SCP-1, kindly provided by Prof. Matthias Schieker [27], was used as osteoprogenitor cells. SCP-1 cells were cultured (37 °C, 5% CO_2_, humidified atmosphere) in MEMα Medium supplemented with 5% FCS. The medium was changed twice a week. Cells were sub-cultured when 80%–90% confluence was reached to prevent spontaneous differentiation.

SaOS-2 cells (DSMZ) were used representative of osteogenic cells. SaOS-2 cells were cultured in RPMI 1640 Medium supplemented with 5% FCS (37 °C, 5% CO_2_, humidified atmosphere). Cells were sub-cultured at 80–90% confluence.

#### 2.3.2. Cell Seeding on the Scaffolds

The medium was thoroughly aspirated from the pre-conditioned scaffolds. Then, one scaffold was placed centrally in each cavity of a 48-well-plate for cell seeding.

Viable THP-1 cells were counted with the trypan blue exclusion method. The required number of cells was spun down (600× *g* for 10 min) and resuspended with culture medium containing 200 nM PMA (phorbol 12-myristate 13-acetate) to obtain a concentration of 5.3 × 10^6^ cells/mL.

SCP-1 and SaOS-2 cells were detached from the culture flask with Trypsin/EDTA. Viable cells were counted with the trypan blue exclusion method. The required number of cells were spun down (600× *g* for 10 min) and resuspended with culture medium to obtain a concentration of 1.3 × 10^6^ cells/ml (SCP-1 cells) and 2.7 × 10^6^ cells/ml (SaOS-2 cells), respectively.

Fifteen microliters of this cell suspension was dripped centrally on top of each scaffold, to obtain seeding densities of 8 × 10^4^ cells/scaffold for THP-1 cells, 2 × 10^4^ cells/scaffold for SCP-1 cells, and 4 × 10^4^ cells/scaffold for SaOS-2 cells. After an initial incubation of 4 h in (37 °C, 5% CO_2_, humidified atmosphere), 505 µL of the respective cell culture medium was carefully added. To allow complete adherence, the specimens were incubated for 24 h (37 °C, 5% CO_2_, humidified atmosphere).

#### 2.3.3. Osteogenic Differentiation of SCP-1 Cells

To induce osteogenic differentiation of SCP-1 cells, the culture medium was thoroughly aspirated and replaced by osteogenic differentiation medium (MEMα medium supplemented with 1% FCS, 200 μM L-ascorbate-2-phosphate, 5 mM β-glycerol-phosphate, 25 mM HEPES, 1.5 mM CaCl_2_, and 100 nM dexamethasone) [28]. 3D-cultures were cultured at 37 °C (5% CO_2_, humidified atmosphere) with complete medium changes on days 1, 4, 7, and 11 of culture.

#### 2.3.4. Osteogenic Maturation of SaOS-2 Cells

For maturation of SaOS-2 cells, the culture medium was replaced by osteogenic medium (RPMI 1640, 2% FCS, 200 μM L-ascorbic acid 2-phosphate, 5 mM β-glycerol phosphate, 25 mM HEPES, 1.5 mM CaCl_2_, and 5 μM cholecalciferol) [29]. 3D-cultures were maintained at 37 °C (5% CO_2_, humidified atmosphere). The osteogenic medium was replaced on days 1, 4, 7, and 11 of culture.

#### 2.3.5. Osteoclastic Differentiation of THP-1 Cells

To induce osteoclastic differentiation of THP-1 cells [30], the culture medium was carefully aspirated and replaced by 390 µL of fresh and 130 µL conditioned medium from maturing SaOS-2 cells differentiated in a T175 cell culture flask (Section 2.3.2). 3D-cultures were incubated at 37 °C (5% CO_2_, humidified atmosphere), and the medium was completely changed on days 1, 4, 7, and 11 of culture.

### 2.4. Functional Testings

#### 2.4.1. Live–Dead-Staining

Viable cells were visualized using the cell-permeable non-fluorescent calcein-AM dye, which is converted into green fluorescent calcein by esterases in the cell cytoplasm. Nuclei were counterstained with Hoechst 33,342 (blue fluorescent when intercalated into DNA) [7]. The nuclei of dead cells were visualized using the cell-impermeable Ethidium-bromide, which, upon intercalation into DNA, gives a red fluorescent signal. Scaffolds with and without attached cells were washed once with PBS before incubation with the staining solution (plain culture medium supplemented with 2 µM calcein-AM, 3.5 µM Hoechst 33,342, and 25 µM Ethidium-bromide). After 15 min, constructs were washed two times with PBS, and fluorescence signals were immediately measured with the fluorescence microscope (Evos Fl).

#### 2.4.2. Mitochondrial Activity (Resazurin Conversion)

To determine the mitochondrial activity of the cells attached to the scaffolds, the constructs were washed once with PBS and transferred to new 48-well-plates. Scaffolds were immediately covered with 500 µL of a 0.0025% resazurin solution (in plain culture medium). Scaffolds without cells were used as background control. After incubation for 2 h at 37 °C, the produced resorufin was measured by the fluorescence at 544 nm/590–10 nm using the Omega Plate Reader (BMG Labtech, Ortenberg, GER). To do so 2 × 100 µL of each sample was transferred into cavities of a 96-well-plate [7].

#### 2.4.3. Quantification of Total DNA

To isolate the total DNA of cells differentiated on the scaffolds, samples were washed once with PBS. Scaffolds without cells were used as background control. Scaffolds were incubated in 250 µL of 50 mM hot (98 °C) NaOH for 15 min. Then, the samples were frozen at −80 °C for, at least, 24 h. After thawing at 60 °C for 20 min, 250 µL of a 100 mM Tris buffer (pH = 8.0) was added to each sample to neutralize pH. These samples were centrifuged at 1000× *g* for 10 min to remove impurities. DNA concentration of supernatants was determined photometrically using the LVIS plate and the Omega Plate Reader (BMG Labtech) [7].

#### 2.4.4. Dot Blot Analysis

Dot blot was performed to detect specific proteins in the culture supernatants. Briefly, 60 µL cell culture supernatant was applied on a wet nitrocellulose membrane with the help of a dot blotter (Carl Roth). The transfer of proteins was confirmed by Ponceau staining. Membranes were blocked with 5% BSA in TBS-T for 1 h, followed by overnight incubation at 4 °C with primary antibodies (diluted 1:1000 in TBS-T) against the target proteins. Target proteins and their specific antibodies are summarized in Table 2. The next day, after washing 3 times with TBS-T for 15 min, the membranes were incubated with the corresponding peroxidase-labeled secondary antibodies (1:5000 in TBS-T/Santa Cruz Biotechnology) for 2 h. For signal development, membranes were incubated for 1 min with ECL substrate solution. Chemiluminescent signals, detected by a CCD camera (INTAS, Göttingen, Germany), were quantified using the ImageJ software [7].

#### 2.4.5. Changes in the Scaffolds’ Stiffness and Mineral Content

After 14 days of differentiation, the colonized scaffolds were fixed with 3.7% formalin for 15 min. After washing with PBS, the scaffolds were analyzed for stiffness and mineral content, as described above (Section 2.2.4 and Section 2.2.5).

### 2.5. Statistical Analysis

Results are presented as violin plots (median and quartiles), floating symbols, or bar diagrams (mean ± 95% confidence interval). Each experiment was performed 3 times (N = 3), with, at least, three replicates (N ≥ 3). Statistical analyses were performed using the GraphPad Prism Software (GraphPad, El Camino Real, San Diego, CA, USA). Data obtained from the two scaffolds were compared by non-parametric Mann–Whitney U-test. A *p*-value below 0.05 was considered statistically significant.

## 3. Results

### 3.1. GEL Scaffolds Have Larger Pores Than PRP Scaffolds

**Figure 2:**
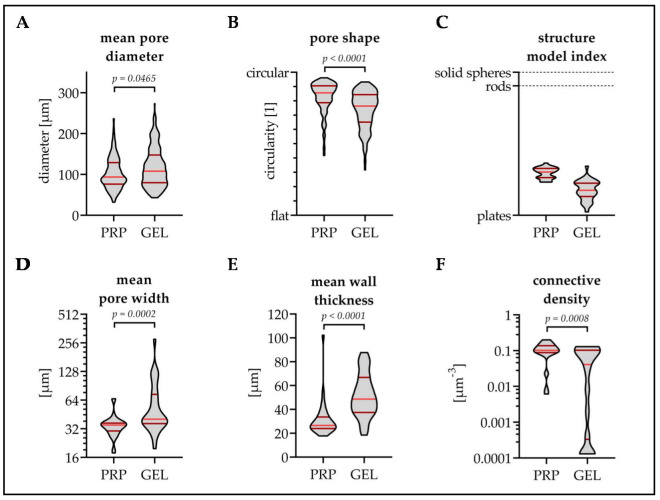
Optical analyses of the pore structure—comparison of the PRP and gelatin (GEL) scaffolds. Scaffold walls were stained with SRB, and the resulting fluorescent images (40× magnification) were analyzed using the ImageJ software. (**A**) Mean pore diameter and (**B**) pore shape were determined manually using the “polygon selection” tool. More detailed analyses were performed using the plugin “BoneJ” which provided information on (**C**) the shape of the walls determined by the “structure model index” after Hildebrand and Rüegsegger; (**D**) the mean pore width determined by the “trabecular separation”; (**E**) the mean wall thickness determined by the “trabecular thickness”; and (**F**) the resulting “connective density”. For each scaffold, 30 images were analyzed. Non-parametric Mann–Whitney U-test was used for data comparison.

Both scaffolds were compared regarding their pore structures. Fluorescent images from SRB stained scaffold walls were analyzed using the ImageJ software. The mean pore diameter of the GEL scaffolds (117.6 ± 22.0 µm) was larger than the mean pore diameter of the PRP scaffolds (102.6 ± 24.4 µm/*p* = 0.0465, Figure 2A). The pores of the PRP scaffolds had a more roundish shape than the ones of the GEL scaffolds (Figure 2B). The structure model index revealed that the interconnecting walls of both scaffolds had a plate-like shape (Figure 2C). More detailed analyses using the plugin “BoneJ” confirmed that the mean pore width of the GEL scaffolds (63.1 ± 51.7 µm) was significantly larger than that of the PRP scaffolds (35.1 ± 10.1 µm/*p* = 0.0002, Figure 2D). The mean wall thickness of the GEL scaffolds (51.8 ± 18.9 µm) was also significantly larger than that of the PRP scaffolds (31.7 ± 15.5 µm/*p* < 0.0001, Figure 2E). This resulted in a higher connective density of the PRP scaffolds (0.104 ± 0.052 µm^−3^) when compared to the GEL scaffolds (0.053 ± 0.052 µm^−3^/*p* = 0.0008, Figure 2F).

### 3.2. PRP Scaffolds Have A Higher Porosity and Stiffness Than GEL Scaffolds

**Figure 3:**
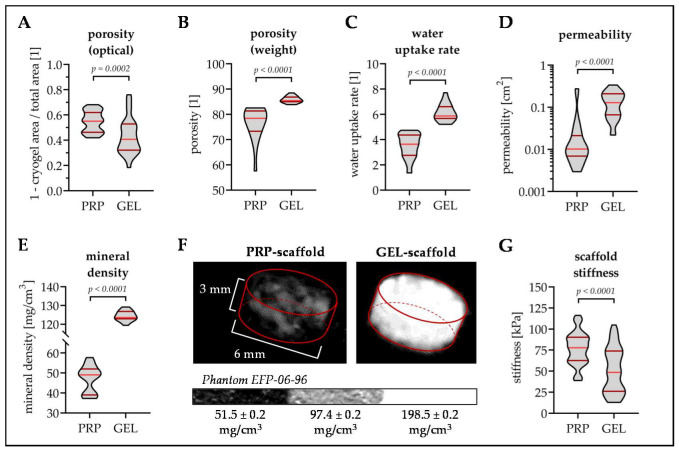
Physical characteristics of the PRP and GEL scaffolds. (**A**) Porosity of the two scaffolds was optically determined (fluorescent images with 40× magnification) using the plugin “BoneJ” of the ImageJ software (“volume fraction”). In addition, (**B**) the porosity and (**C**) the water uptake rate was determined via the dry and wet weight of the scaffolds. (**D**) The permeability of the two scaffolds was determined based on the law of Darcy. (**E**) The mineral content of the two scaffolds was determined by quantitative computed tomography (CT). (**F**) Showing representative 3D reconstructions of the CT scans. (**G**) Scaffold stiffness was determined by cyclic compressions using a material testing machine. For each scaffold, at least, 15 individual measurements were taken for analysis. Non-parametric Mann–Whitney U-test was used for data comparison.

Porosity was determined both by analysis of the fluorescent images (Figure 3A) and by weighing the scaffolds in dry and wet state (Figure 3B). In the optical analysis, where porosity was described in the hydrated state, PRP scaffolds had a significantly higher porosity (54.7 ± 8.3%) than GEL scaffolds (43.1 ± 14.2%, *p* = 0.0002). By weighing scaffolds, where porosity was described in the dry state, GEL scaffolds had a significantly higher porosity (85.9 ± 1.3%) than PRP scaffolds (76.5 ± 6.5%, *p* < 0.0001). The water uptake rate (Figure 3C) and permeability (Figure 3D) were significantly higher for GEL scaffolds (6.1 ± 0.1 and 0.14 ± 0.09 cm^2^) than for PRP scaffolds (3.5 ± 1.0 and 0.03 ± 0.06 cm^2^, *p* < 0.0001).

Quantitative CT scans revealed that mineral content was significantly higher in GEL scaffolds (124.4 ± 2.6 mg/cm^3^) than in PRP scaffolds (47.2 ± 6.8 mg/cm^3^, *p* < 0.0001, Figure 3E,F). Instead, the scaffold stiffness was significantly higher for PRP scaffolds (76.8 ± 19.2 kPa) than for GEL scaffolds (51.1 ± 26.5 kPa, *p* < 0.0001, Figure 3G).

### 3.3. Osteogenic Cells Show Better Cell Attachment and Faster Proliferation on GEL Than on PRP Scaffolds

**Figure 4:**
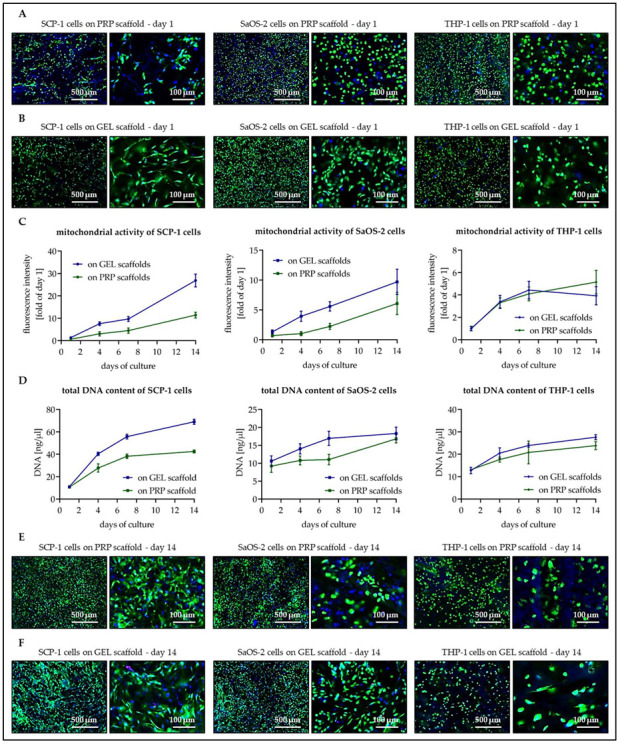
Viability of the different cell types over time on the two scaffold types. Twenty thousand SCP-1 cells, 4 × 10^4^ SaOS-2 cells, and 8 × 10^4^ THP-1 cells were plated per scaffold. (**A**,**B**) Twenty-four hours after seeding, (**E**,**F**) as well as after 14 d of differentiation, cells on the scaffolds ((**A**,**E**) PRP scaffolds and (**B**,**F**) GEL scaffolds) were visualized by live–dead staining: green cytoplasm in all living cells (calcein-AM), blue nuclei in all cells (Hoechst 33342) and red nuclei in all dead cells (Ethidium bromide). On days 1, 4, 7, and 14 of differentiation (**C**) mitochondrial activity was determined by resazurin conversion and (**D**) the amount of total DNA was determined by absorbance measurement. Each experiment was performed three times (N = 3) in triplicates (n = 3).

Scaffolds were colonized with SCP-1 cells (osteogenic precursor cells), SaOS-2 cells (mature osteoblasts), or THP-1 cells (osteoclastic precursor cells). In preliminary experiments, the optimal cell numbers for a confluent seeding were determined to be 2, 4, and 8 × 10^4^ cells per scaffold, respectively (data not shown). Twenty-four hours after seeding, the density of the osteogenic cells was slightly lower on PRP scaffolds (Figure 4A) than on GEL scaffolds (Figure 4B). On both scaffold types, SCP-1 cells settled mainly on the surface of the scaffold and revealed a stretched morphology. SaOS-2 cells, on the contrary, conserved a roundish morphology, which enabled the cells to penetrate sub-surface areas of both scaffolds. The seeding pattern of THP-1 cells was comparable on both scaffolds after 24 h. Similar to SaOS-2 cells, THP-1 cells showed a roundish morphology and also settled in sub-surface areas of the scaffolds.

This impression was confirmed by the mitochondrial activity of the cells on the scaffolds (Figure 4C). The first day after seeding, the osteogenic cells had a slightly higher mitochondrial activity on GEL scaffolds when compared to PRP scaffolds. With time mitochondrial activity of SCP-1 cells increased more rapidly when cultured on GEL scaffolds than on PRP scaffolds (slopes _days 1–14_: 1.94 ± 0.09 vs. 0.83 ± 0.05, *p* < 0.001). For SaOS-2 cells, this effect was less pronounced (slopes _days 1–14_: 0.63 ± 0.06 vs. 0.43 ± 0.05, *p* = 0.013). On both scaffolds types, a steady increase in mitochondrial activity was observed. This was different for THP-1 cells, which showed no significant difference in mitochondrial activity when cultured on GEL scaffolds or PRP scaffolds (slopes _days 1–7_: 0.58 ± 0.07 vs. 0.51 ± 0.05, *p* = 0.470) and reached a plateau after 7 d of culture.

A similar trend was observed with the total DNA content (Figure 4D). The biggest increase in total DNA content was observed with SCP-1 cells on GEL scaffolds followed by SCP-1 cells on PRP scaffolds (slopes: 4.1 ± 0.2 vs. 2.2 ± 0.2, *p* < 0.001). The increase in total DNA content of SaOS-2 cells (slopes: 0.57 ± 0.09 vs. 0.58 ± 0.07, *p* = 0.915) and THP-1 cells (slopes: 1.05 ± 0.09 vs. 0.79 ± 0.14, *p* = 0.108) was comparably on the two scaffolds.

Live–dead-staining of the cells on day 14 of culture (Figure 4E,F) revealed a high viability of cells on both scaffolds. Only on the GEL scaffolds were a few dead cells found, mostly in inner parts of the scaffold. The highest cell density was observed with SCP-1 cells. Especially on GEL scaffolds, it was barely possible to identify single SCP-1 cells on the scaffolds. SaOS-2 and THP-1 cells also showed a high density on the scaffolds. These cell types changed towards a more stretched morphology when compared to day 1.

### 3.4. Levels of Osteoblast and Osteoclast Markers in Culture Supernatant

**Figure 5:**
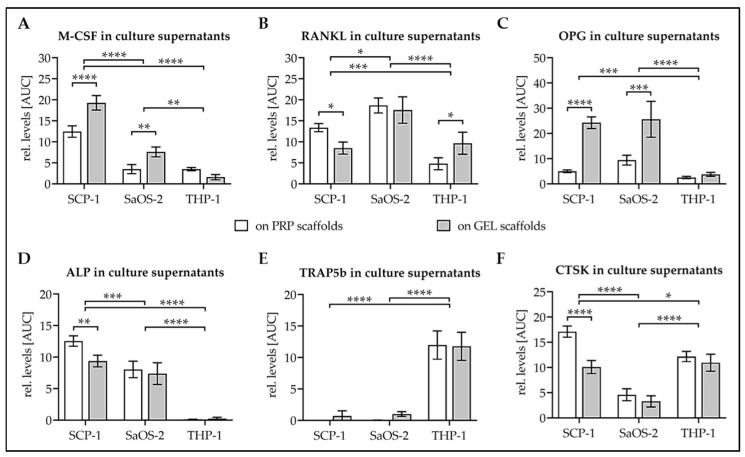
Factors secreted into the culture medium by the different cells on the two scaffolds. Twenty thousand SCP-1 cells, 4 × 10^4^ SaOS-2 cells, and 8 × 10^4^ THP-1 cells were seeded on each scaffold and differentiated for 14 d. On days 4, 7, and 14, conditioned medium (72 h) was collected. Dot blot analysis was performed to determine relative levels of (**A**) macrophage colony-stimulating factor = M-CSF, (**B**) receptor activator of nuclear factor kappa-Β ligand = RANKL, (**C**) osteoprotegerin = OPG, (**D**) alkaline phosphatase = ALP, (**E**) tartrate-resistant acidic phosphatase 5b = TRAP5b, and (**F**) cathepsin K = CTSK. Each experiment was performed three times (N = 3) in triplicates (N = 3). AUC = area under the curve, summarizing the 14 days of differentiation. Two-way ANOVA was used for data comparison between groups. * *p* < 0.05, ** *p* < 0.01, *** *p* < 0.001, and **** *p* < 0.0001 as indicated.

To determine cell function, levels of characteristic proteins released into the culture supernatant were determined. As regulators of osteoclastogenesis macrophage colony-stimulating factor (M-CSF), receptor activator of nuclear factor kappa-Β ligand (RANKL), and osteoprotegerin (OPG) were detected. M-CSF levels were higher in culture supernatants from SCP-1 cells than SaOS-2 cells and barely detectable in culture supernatants from THP-1 cells. M-CSF levels were significantly higher in culture supernatants when the osteogenic cell lines were differentiated on GEL scaffolds (Figure 5A). RANKL levels were higher in culture supernatants from SaOS-2 cells than SCP-1 cells. THP-1 cells, which were differentiated with conditioned SaOS-2 medium, showed the lowest levels of RANKL (Figure 5B). OPG levels were comparable between culture supernatants from SCP-1 cells and SaOS-2 cells and barely detectable in culture supernatants from THP-1 cells. Interestingly, OPG levels were significantly higher in culture supernatants of osteogenic cells differentiated on GEL scaffolds (Figure 5C).

Alkaline phosphatase (ALP) was detected as an early osteoblast marker. Over the 14 d of differentiation, ALP levels were higher in SCP-1 cultures than in SaOS-2 cultures; however, with a timely difference. ALP levels were very high early and dropped rapidly with differentiation of SaOS-2 cells. In the case of SCP-1 cells, ALP levels continuously increased (data not shown). ALP was barely detectable in culture supernatants of differentiating THP-1 cells (Figure 5D). Inversely, the osteoclast marker tartrate-resistant acidic phosphatase 5b (TRAP5b) was close to the detection limit in culture supernatants of SCP-1 cells and SaOS-2 cells. TRAP5b levels were only detectable in culture supernatants of differentiating THP-1 cells, with TRAP5b levels increasing with differentiation time in these cultures (Figure 5E). Levels of the other commonly used osteoclast marker cathepsin K (CTSK) also increased with time in culture supernatants of THP-1 cells, resulting in significantly higher levels than in culture supernatant of SaOS-2 cells over the entire course of differentiation. Interestingly, over the entire 14 d of differentiation, the highest levels of CTSK were detected in culture supernatants of SCP-1 cells (Figure 5F).

### 3.5. Culture with the Cells Alters Stiffness and Mineral Content of the Scaffolds

**Figure 6:**
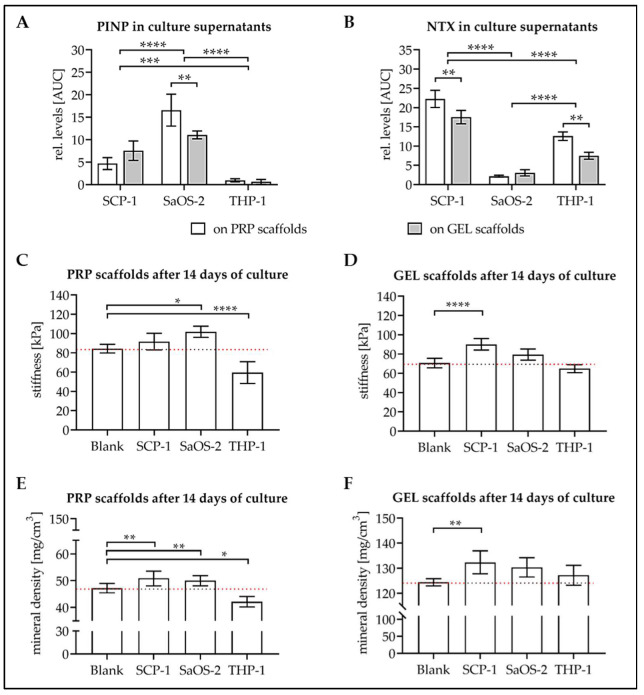
Changes in scaffold matrix after 14 days of culture with the different cell types. Twenty thousand SCP-1 cells, 4 × 10^4^ SaOS-2 cells, and 8 × 10^4^ THP-1 cells were seeded on each scaffold and differentiated. On days 4, 7, and 14, conditioned medium (72 h) was collected. Dot blot analysis was performed to determine relative levels of (**A**) PINP = procollagen type I N-terminal propeptide and (**B**) NTX = collagen-type I N-telopeptide. AUC = area under the curve, summarizing the 14 d of differentiation. On day 14 (**C**,**D**) stiffness and (**E**,**F**) mineral content of (**C**,**E**) PRP scaffolds and (**D**,**F**) GEL scaffolds were determined. Each experiment was performed three times (N = 3), at least, in triplicates (N ≥ 3). Two-way ANOVA or non-parametric Friedman test was used for data comparison between groups. * *p* < 0.05, ** *p* < 0.01, *** *p* < 0.001, and **** *p* < 0.0001 as indicated.

To determine alterations in the scaffold matrix induced by the different cell types, proteins formed during type I collagen formation (PINP = procollagen type I N-terminal propeptide) and degradation (NTX = collagen-type I N-telopeptide) were detected in the culture supernatant. PINP levels were highest in culture supernatants from SaOS-2 cells, well detectable in culture supernatants of SCP-1 cells, and barely detectable in culture supernatants from THP-1 cells. NTX levels were similar to levels of the CSTK, which represents the major collagenase in osteoclasts. They were constantly high in culture supernatants of SCP-1 cells but increased with differentiation time in culture supernatants of THP-1 cells. Over the entire course of differentiation, NTX levels were highest in culture supernatants of SCP-1 cells, followed by THP-1 cells. Culture supernatants of SaOS-2 cells showed only very low levels of NTX (Figure 6B).

After 14 d of culture, scaffolds were again analyzed for stiffness and mineral content. PRP scaffolds showed significantly increased stiffness when cultured with SaOS-2 cells (Δ_mean_ = 18 kPa, *p* = 0.0103) and decreased stiffness when cultured with differentiated THP-1 cells (Δ_mean_ = 25 kPa, *p* < 0.0001, Figure 6C). GEL scaffolds, on the contrary, showed significantly increased stiffness only when cultured with differentiating SCP-1 cells (Δ_mean_ = 20 kPa, *p* < 0.0001, Figure 6D).

In line with this, the mineral content of PRP scaffolds was significantly increased when cultured with SCP-1 cells (Δ_mean_ = 3.6 mg/cm^3^, *p* = 0.0089) and SaOS-2 cells (Δ_mean_ = 2.8 mg/cm^3^, *p* = 0.0021). The mineral content was significantly decreased when PRP scaffolds were cultured with differentiated THP-1 cells (Δ_mean_ = 5.1 mg/cm^3^, *p* = 0.0408, Figure 6E). Relatively seen, changes in mineral content were less pronounced with GEL scaffolds, which have a significantly higher basal mineral content. Only the culture with SCP-1 cells could significantly increase the mineral content of the GEL scaffolds (Δ_mean_ = 8.8 mg/cm^3^, *p* = 0.0089, Figure 6F).

## 4. Discussion

Bone tissue engineering aims at producing biologically active bone material, for example, for transplantation into large bone defects. These constructs ideally consist of the patients’ own cells on scaffolds mimicking the three-dimensional environment of bone. Therefore, the 3D carriers should favor osteogenic differentiation of the applied cells either directly or by secretion of paracrine factors [8]. For bone tissue engineering, a large variety of synthetic bioceramics, as well as freeze-dried scaffolds, exist [31,32,33,34]. We recently described the influence of different HEMA-based cryogels with different protein additives on osteogenic characteristics of adipose-derived mesenchymal stem cells (MSCs), with the most promising results obtained by the addition of human platelet-rich plasma (PRP) and rat tail collagen [7]. However, the use of these cryogels raised concerns, as well-known toxins are required for their production, although these substances are nontoxic in the interconnected solid form, and residuals may effectively be removed by an adequate cleaning protocol [7,21,22]. However, during the in vivo degradation of HEMA-based carriers, toxic methacrylic acid may form [23]. To minimize the risk for toxicity, we here generated simplified cryogels containing only cold-water fish gelatin (GEL). Cryogels based on this natural material are thought to be biologically converted when applied in vivo [11]. Non-covalent aggregations of gelatin are easily dissolved at temperatures higher than 30–35 °C, thereby destroying the physical network. Therefore, gelatin was covalently crosslinked by glutaraldehyde, inducing the coupling of the carboxyl and amino groups to form stable amide bonds, which, in turn, increases the stability and mechanical properties of the resulting cryogels [35]. Collagen is the main organic matrix of bone, which, when hydrolyzed, forms gelatin [36]. Although collagen- or gelatin-based cryogels are closely related to the organic bone matrix, their physical characteristics are often too soft to represent bone tissue [37]. Therefore, the current approached included the formation of composite materials, e.g., the inclusion of hydroxyapatite within the cryogel matrix [37]. The resulting composite materials often have superior physical characteristics, e.g., increased stiffness or pore size [19].

Hydroxyapatite is the major mineral component of bone. Therefore, scaffolds containing hydroxyapatite are often described to favor cell attachment, proliferation, and osteogenic differentiation [31,38]. We have shown that replacing incorporated insoluble hydroxyapatite by externally crystalized calcium phosphates increased both pore size and stiffness in our initial PRP scaffolds [7]. However, this method was not applicable for the GEL scaffolds, as it induced an instant polymerization of the material preventing pore formation. In these scaffolds, insoluble hydroxyapatite was incorporated within the cryogel matrix in large amounts to prevent sedimentation and, consequently, gradient formation, as observed before [7]. The resulting GEL scaffolds had a significantly higher mineral content but lower stiffness than the PRP scaffolds, which had a mean stiffness close to the stiffness of collagenous bone [39,40].

The externally crystallized calcium phosphate increased the surface roughness of the PRP scaffolds [7]. It has been shown that rougher surfaces should promote osteogenic differentiation of MSCs [41,42], possibly because of the additional formation of focal adhesions [43]. Furthermore, stiffness of the carrier was reported to affect cell adherence and spreading, as well as proliferation in a cell type-specific manner [44]. In our experiments, the immortalized human MSCs (SCP-1 cells) produced more ALP when differentiated on PRP scaffolds. SCP-1 cells seemed to proliferate faster on GEL scaffolds than on PRP scaffolds. On day 14, it was barely possible to visualize single SCP-1 cells on these scaffolds. The resulting high cell density, may also suppress the formation of ALP [45]. This may also contribute to the result that the increase in mineral content and stiffness were stronger when SCP-1 cells were differentiated on GEL scaffolds than on PRP scaffolds. In the case of more differentiated SaOS-2 cells, which have a very high basal ALP expression [46], ALP production did not differ when cultured on the two scaffolds. Collagen production (PINP levels), the main organic matrix of bone, was significantly higher in culture supernatants of SaOS-2 cells on PRP scaffolds than on GEL scaffolds. As gelatin is a mixture of proteins obtained by acidic or alkaline hydrolysis of collagen [36], this might contribute, in a kind of negative feedback, to the lower PINP levels observed in culture supernatants of SaOS-2 cells on GEL scaffolds. However, the degradation of collagen (NTX levels) was also higher by cells on PRP scaffolds than on GEL scaffolds. It remains to be discussed whether the collagen formed by the cells themselves can be more easily digested than the chemically modified and covalently crosslinked one in the GEL scaffolds.

Furthermore, the basal scaffold stiffness and mineral content may affect the observed results. It has been proposed that a carrier stiffness larger than 60 kPa, which holds true for our PRP scaffolds, induced expression of osteogenic transcription factors and marker genes [39,47]. This difference was observed only for RANKL and ALP production by SCP-1 cells, not by SaOS-2 cells, in our experiments. Furthermore, ALP activity is reported to be increased when cells are cultured on rougher surfaces [48], e.g., on our PRP scaffolds. Interestingly, M-CSF and OPG production was significantly increased by both osteogenic cell lines when differentiated on GEL scaffolds, which might be triggered by the lower stiffness and/or the higher mineral content in these scaffolds. M-CSF was produced in larger amounts by SCP-1 cells than by SaOS-2 cells independent of the 3D matrix used. However, differentiation of the cells on the GEL scaffold seemed to induce the production of M-CSF, a well-known inducer of osteoclastogenesis [49]. It has been shown that increased crystallinity of the culture surface suppressed expression of M-CSF in rat calvarial cells [50]. This may explain why, on the externally crystalized PRP scaffolds with the rougher surface, the M-CSF production by these cells was lower. OPG, as an indirect inhibitor of osteoclastogenesis, is directly associated with bone stiffness [51]. An increase in hydrogel stiffness was shown to be associated with an increased expression of ALP and OPG [52]. Furthermore, in the same model, expression of ALP and OPG decreased again with an increasing degree of crosslinking and thus decreased porosity [52]. In addition, OPG expression was reported to increase with increasing surface roughness [53]. These examples show that mineral content, surface roughness, stiffness, and porosity of the scaffolds all affect the function of cells on the scaffolds. Although GEL scaffolds had significantly larger pores than PRP scaffolds, the connective density was lower in these scaffolds. This may be attributed to the high water storage capacity of gelatin [35], resulting in increased wall thickness in GEL scaffolds in hydrated states, as observed during culture. This was also observed in our results, where GEL scaffolds with the higher water uptake capacity showed higher porosity in the dry state but lower porosity in the hydrated state when compared with PRP scaffolds. Transferring these observations onto our model would favor the expression of ALP and OPG on the PRP scaffolds, not on the GEL scaffolds. Overall, a pore size between 20 µm and 300 µm is recommended to support osteogenic differentiation in bone tissue engineering approaches [31]. Both our scaffolds lie within this range. However, other studies propose a minimum pore size of 100 µm for successful ingrowth of bone cells into scaffolds [54,55]. This is supported by our study, which showed better ingrowth of the cells into the GEL scaffolds with the larger pores. Together with the fact that this scaffold was based on a natural polymer, this might be an advantage, when considering in vivo application. The bigger pores facilitated the ingrowth of the cells within the carrier material and, consequently, a more rapid cell-induced conversion of the scaffold material in vivo. A limitation may be the incorporation of insoluble hydroxyapatite within the scaffold. A calcium phosphate that is more easily biologically converted may be of advantage [56], but requires further research [37].

As representatives of the osteoclast lineage, myeloid THP-1 cells were differentiated to osteoclastic cells using conditioned medium of differentiating SaOS-2 cells, which produce M-CSF and RANKL required to induce osteoclastogenesis [57]. RANKL production might be even supported by the replacement of dexamethasone with cholecalciferol in the medium [58]. As the THP-1 cells were differentiated with the same medium, cells were exposed to the same exogenous factors. Therefore, observed effects can be attributed to the scaffolds the cells were differentiated on. In contrast, the osteogenic cells’ adherence to THP-1 cells to the two scaffolds was comparable. After 14 days of differentiation, cell density was still comparable on both scaffolds. Likewise, the production of osteoclast markers TRAP5b and CTSK was comparable between the two conditions, showing that THP-1 cells can gain some osteoclastic features. Although, there was no significant difference in the levels of the collagenase CTSK, collagen degradation (NTX levels) was significantly stronger by THP-1 cells on PRP scaffolds than on GEL scaffolds. Similarly, matrix stiffness and mineral content decreased significantly only when THP-1 cells were differentiated on PRP scaffolds, supporting the use of PRP scaffolds, when it comes to investigating osteoclast function of differentiated THP-1 cells. It has been shown that osteoclast fusion is strongly dependent on the surface the cells attach to [59]. For example, only the presence of both organic and inorganic bone matrix induces the expression of Annexin A8 required for the initial cell fusion [59]. Therefore, it is feasible, that PRP scaffolds, with the externally crystallized calcium phosphate better represent a bone matrix that induces osteoclastogenesis [60], and may be a suitable carrier for a stable in vitro system for bone metabolism investigating the function of osteoblasts and osteoclasts. Furthermore, the crystals lying on the surface of the PRP scaffolds may be more easily accessible for the cells than the insoluble hydroxyapatite crystals incorporated within the GEL scaffolds, at least, partly explaining the observed decrease in mineral content in the PRP scaffolds after 14 d of culture with differentiating THP-1 cells. Another explanation may be due to the calcium-sensing ability of the osteoclasts themselves. The balance between matrix and soluble calcium is thought to regulate osteoclast activity and apoptosis [61].

## 5. Conclusions

In summary, our data showed that both scaffolds were compatible with the tested cell lines. While osteogenic SCP-1 cells and SaOS-2 cells attached better to the GEL scaffold than the PRP scaffold, THP-1 cells had no preference in attaching to either of the two scaffolds. In addition, GEL scaffolds seemed to induce proliferation, especially of the SCP-1 cells. Besides cell attachment and survival, each of the two scaffolds presents powerful advantages. On the one hand, the PRP scaffolds had a higher stiffness and a rougher mineralized surface, which enabled degradation of the matrix by THP-1 derived osteoclastic cells. On the other hand, the GEL scaffolds had a higher mineral content closer to the bone matrix and larger pores, which enabled the infiltration of cells with time. The simplified way of production favors the use of GEL scaffolds, however, the possibility to alter mineral content and stiffness, as was seen mainly with PRP scaffolds, may be decisive when it comes to choosing a scaffold for in vitro assay systems, while biological degradation may be favorable for bone tissue engineering when considering in vivo application.

## Figures and Tables

**Table 1:** Common raw materials for cryogel production. Information obtained from [11]. The abbreviations were listed in Section Abbreviations.

Monomers	Cross-linker	Starter
alginate	G:M ratio	Ca^2+^, Mg^2+^, etc.
gelatin	Glutaraldehyde	
collagen		
fibrinogen		
serum albumin		
hyaluronic acid		
chitosan		
acrylamide (AAm) and its derivatives	N,N-methylene(bis) acrylamide (BAAm) or methyl-methacrylate (MMA) or poly-ethylene glycol diacrylate (PEGDA) or biodegradable cross-linkers	ammonium persulfate (APS) + N,N,N,N-tetramethyl- ethylenediamine (TEMED)
acrylic acid (AAc)		
2-hydroxyethyl methacrylate (HEMA)		
ethylene glycol diglycidyl ether (EGDE)		
1,4-butanediol diglycidyl ether (BDDE)		
2-acrylamido-2-methylpropane sulfonic acid sodium salt (AMPS)		

**Table 2:** Dot blot targets and conditions.

Target Protein	Role	Species	Order #	Company
ALP	early osteogenic marker	goat	sc-23430	Santa Cruz, Heidelberg, GER
Cathepsin K	osteoclast marker	mouse	sc-48353	Santa Cruz, Heidelberg, GER
M-CSF	inducer for osteoclastogenesis	rabbit	500-P44	Peprotech, Hamburg, GER
NTX	collagen type I degradation	rabbit	PAA639hu01	Cloud-Clone, Aachen, GER
OPG	inhibitor for RANKL	rabbit	500-P149	Peprotech, Hamburg, GER
PINP	collagen type I formation	rabbit	abx131414	Abbexa, Aachen, GER
RANKL	inducer for osteoclastogenesis	mouse	500-M46	Peprotech, Hamburg, GER
TRAP5b	osteoclast marker	mouse	sc-376875	Santa Cruz, Heidelberg, GER

ALP = alkaline phosphatase; M-CSF = macrophage colony-stimulating factor; NTX = collagen-type I N-telopeptide; OPG = osteoprotegerin; PINP = procollagen type I N-terminal propeptide; RANKL = receptor activator of nuclear factor kappa-Β ligand; TRAP5b = tartrate-resistant acidic phosphatase 5b. Abbreviations are additionally listed in Section Abbreviations.

## Abreviations

AAc	acrylic acid
AAm	acrylamide
ALP	alkaline phosphatase
AMPS	2-acrylamido-2-methylpropane sulfonic acid sodium salt
APS	ammonium persulfate
BAAm	N,N-methylene(bis)acrylamide
BDDE	1,4-butanediol diglycidyl ether
CTSK	cathepsin K
EGDE	ethylene glycol diglycidyl ether
GEL	gelatin
HA	hydroxyapatite
HEMA	2-hydroxyethyl methacrylate
M-CSF	macrophage colony-stimulating factor
MMA	methyl-methacrylate
NTX	collagen-type I N-telopeptide
OPG	Osteoprotegerin
PEGDA	poly-ethylene glycol diacrylate
PINP	procollagen type I N-terminal propeptide
PRP	platelet-rich plasma
RANKL	receptor activator of nuclear factor kappa-Β ligand
TEMED	N,N,N,N-tetramethyl-ethylenediamine
TRAP5b	tartrate-resistant acidic phosphatase 5b
